# The Relationship between Vessel Traffic and Noise Levels Received by Killer Whales (*Orcinus orca*)

**DOI:** 10.1371/journal.pone.0140119

**Published:** 2015-12-02

**Authors:** Juliana Houghton, Marla M. Holt, Deborah A. Giles, M. Bradley Hanson, Candice K. Emmons, Jeffrey T. Hogan, Trevor A. Branch, Glenn R. VanBlaricom

**Affiliations:** 1 School of Aquatic & Fishery Sciences, University of Washington, Seattle, WA, United States of America; 2 Conservation Biology Division, Northwest Fisheries Science Center, National Marine Fisheries Service, National Oceanic and Atmospheric Administration, Seattle, WA, United States of America; 3 Wildlife, Fish, & Conservation Biology, University of California Davis, Davis, CA, United States of America; 4 Cascadia Research Collective, Olympia, WA, United States of America; 5 U.S. Geological Survey, Washington Cooperative Fish and Wildlife Research Unit, Seattle, WA, United States of America; Pacific Northwest National Laboratory, UNITED STATES

## Abstract

Whale watching has become increasingly popular as an ecotourism activity around the globe and is beneficial for environmental education and local economies. Southern Resident killer whales (*Orcinus orca*) comprise an endangered population that is frequently observed by a large whale watching fleet in the inland waters of Washington state and British Columbia. One of the factors identified as a risk to recovery for the population is the effect of vessels and associated noise. An examination of the effects of vessels and associated noise on whale behavior utilized novel equipment to address limitations of previous studies. Digital acoustic recording tags (DTAGs) measured the noise levels the tagged whales received while laser positioning systems allowed collection of geo-referenced data for tagged whales and all vessels within 1000 m of the tagged whale. The objective of the current study was to compare vessel data and DTAG recordings to relate vessel traffic to the ambient noise received by tagged whales. Two analyses were conducted, one including all recording intervals, and one that excluded intervals when only the research vessel was present. For all data, significant predictors of noise levels were length (inverse relationship), number of propellers, and vessel speed, but only 15% of the variation in noise was explained by this model. When research-vessel-only intervals were excluded, vessel speed was the only significant predictor of noise levels, and explained 42% of the variation. Simple linear regressions (ignoring covariates) found that average vessel speed and number of propellers were the only significant correlates with noise levels. We conclude that vessel speed is the most important predictor of noise levels received by whales in this study. Thus, measures that reduce vessel speed in the vicinity of killer whales would reduce noise exposure in this population.

## Introduction

Top predators are key components of ecosystems around the globe. Their removal, and the consequent loss of ecological interactions they facilitate, may be detrimental to natural ecosystems [1,2]. The large spatial range required by many top predators leads to competition with humans for space and resources, leaving many in danger of negative anthropogenic interactions [3]. A variety of human interactions, such as exploitation, habitat degradation, and pollution, are known to have negative effects on wildlife populations, while even non-lethal human disturbance, such as wildlife viewing, can be perceived by observed animals as a predation risk and result in energy costs and effects on survival and reproduction [4]. Therefore, it is important to better understand the extent of human use of the environment so that negative consequences on animal populations can be assessed and mitigated.

The potential impacts of human interactions with animals in the marine environment are sometimes difficult to evaluate, because of our inability to see effects on underwater communities. Nevertheless, it is likely that increases in maritime activity and vessel traffic will lead to more harmful impacts such as vessel collision and habitat degradation due to noise pollution. Marine mammals are especially vulnerable to these impacts due to their large size, their requirement to breath at the surface, and life history strategies (e.g., long-lived, delayed reproduction in many cases) [5]. Hearing is important for marine mammals, since sound travels much further in water, whereas light attenuates rapidly. Toothed whales are particularly reliant on echolocation and their acoustic habitat for communication, foraging, and predator detection [6,7], and can be expected to be disproportionately affected by noise pollution.

Since they frequently use densely populated inland waters from central California to southeast Alaska [8], Southern Resident killer whales (*Orcinus orca*; hereafter SRKW) are potentially vulnerable to negative anthropogenic impacts from vessel traffic and ambient noise [8]. Their population was substantially reduced as a result of removals for the aquarium trade in the mid-20th century [8], and then began a slow recovery to 98 individuals by 1995. However, from 1996 to 2001, the population declined by almost 20% for unknown reasons [8]. SRKW were listed as endangered under the U.S. Endangered Species Act in 2005 and a Recovery Plan was developed to determine potential causes for the population decline [8]. Major threats to SRKW recovery were identified as availability and quality of prey, contaminants, and disturbance from vessels and anthropogenic noise.

SRKW utilize calls, clicks, and whistles for navigation, communication, and foraging [9, 10]. Each of the J, K, and L pods (family group) has a distinctive call repertoire and therefore SRKW likely use these vocalizations for group and possibly individual identification [11,12]. Acoustic communication among SRKW individuals is important for group cohesion, cooperative foraging, and social behavior that may involve reproduction [9,10]. Echolocation involves the production of sounds and use of the resulting echo returns to perceive the environment. Echolocation is the primary foraging tool for SRKW [10]. SRKW specialize on many depleted stocks of salmonid species [13,14], so any anthropogenic factor that may limit foraging efficiency could negatively impact the SRKW population.

**Fig 1:**
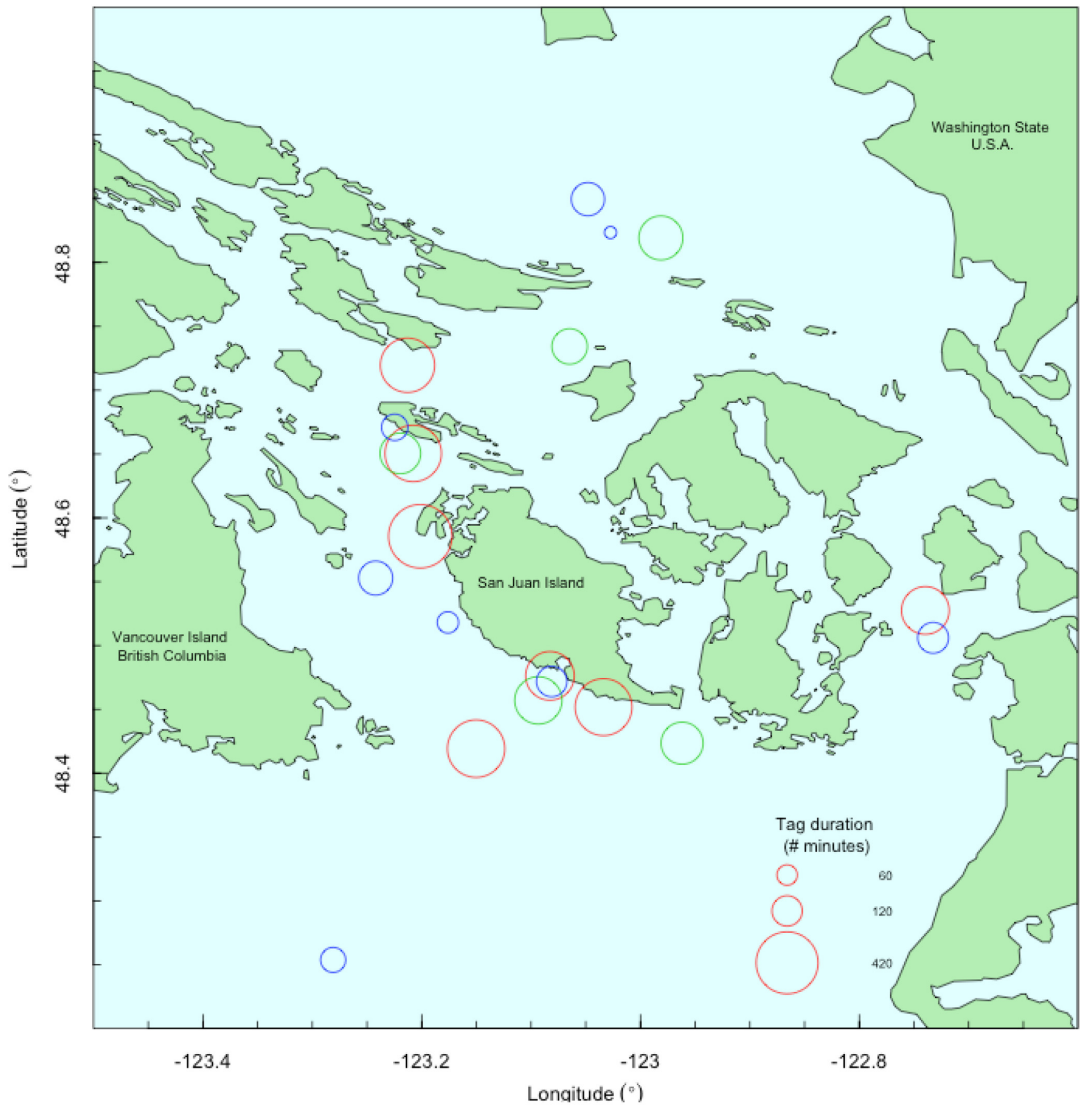
Locations of tag deployments. Locations of tag deployments during which vessel data were collected concurrently (n = 20). The color of the ring corresponds to the year as follows: 2010 –red, 2011 –green, 2012 –blue. The size of the ring depicts the duration of the tag deployment in minutes. The tagged whale travelled beyond the area designated by the ring throughout the deployment period.

SRKW in the Salish Sea (i.e., the inland waters of Washington State and British Columbia; Fig 1) were the focus of this project. The Salish Sea includes critical habitat (as defined by [15]) and core summer habitat of SRKW (as defined by [16]) and is particularly relevant to the impact of vessel traffic as vessel presence has increased dramatically from whale watching, fishing, and shipping [8]. SRKW are the primary focus of a whale watching fleet in the Salish Sea that increased from fewer than 20 commercial boats in the 1980s to roughly 80 boats servicing half a million customers per year by 1998 [17,8], and has remained at this level in recent years [18–20]. Whale watching is now worth more than $70 million annually to the economy in Washington State and British Columbia ([21, 22], S. Russell, NOAA Northwest Fisheries Science Center, pers. comm.), increasing incentives to manage the population to recovery. SRKW in the Salish Sea provide a unique opportunity to study interactions between direct human use of the marine environment and top predators with significant implications for endangered species management.

**Fig 2:**
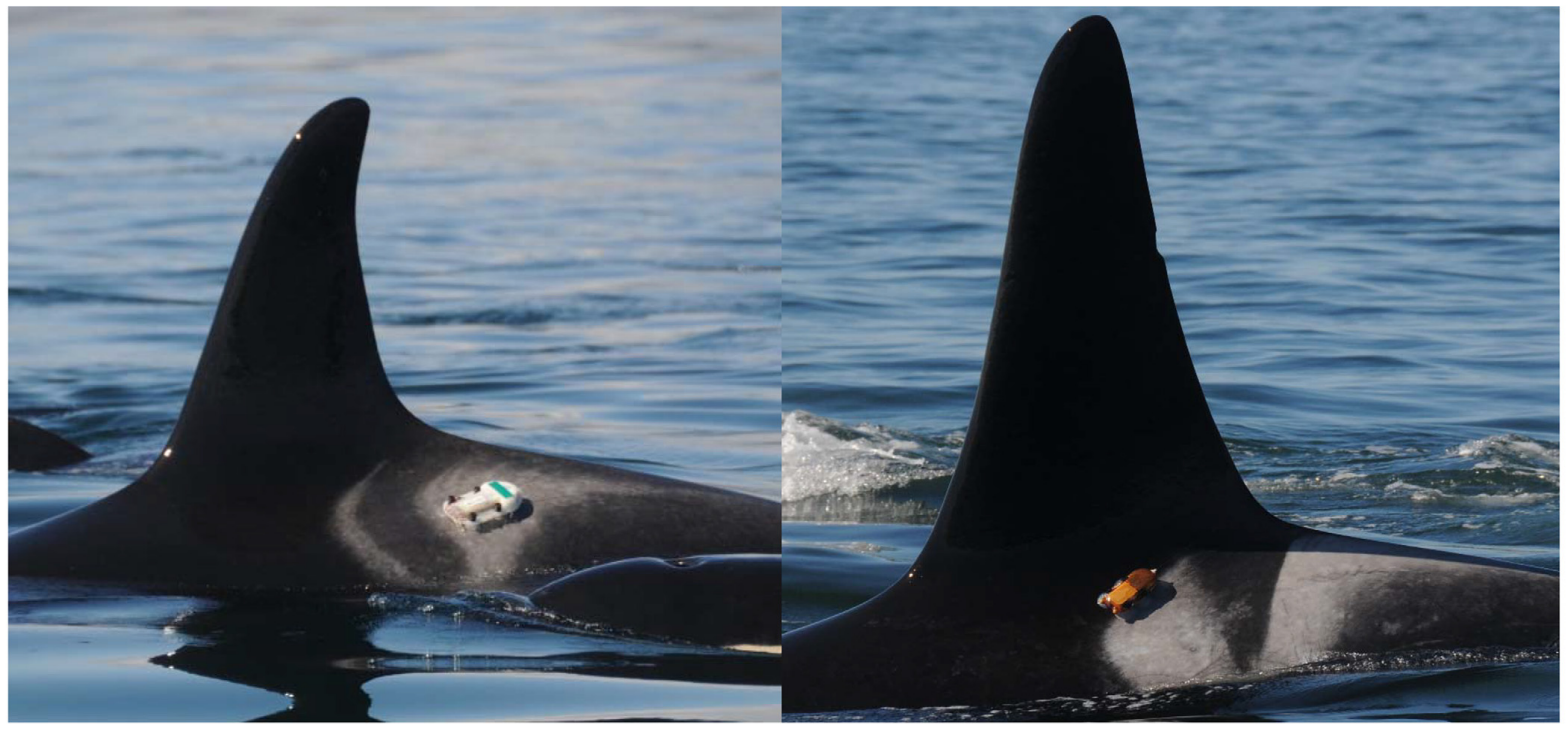
DTAGs on SRKW. Version 2 DTAG on SRKW 9/22/2010 (left) and version 3 DTAG on SRKW 9/17/2012.

SRKW are known to alter their behavioral states in the presence of vessels [23–27]. Increased environmental noise also leads to vocal modification by SRKW [28–31]. None of the previous studies have measured the ambient noise that individual killer whales actually receive nor precisely measured the vessel traffic characteristics surrounding the whales. To address this limitation, digital acoustic recording tags (DTAGs; Fig 2) and laser positioning systems were utilized concurrently in a large, collaborative project. The larger project aims to understand the effects of vessels and associated noise on SRKW behavior, and the current study is the first phase toward this goal. DTAGs have been used on a variety of cetacean species to examine vocal and movement behavior [32,33], but few have utilized ambient noise recordings for inferences regarding the changes in the acoustic environment a whale experiences. The laser positioning system allows for a more accurate measure of vessel presence by determining the precise position of the tagged whale and any vessel within 1000 meters and recording vessel characteristics (e.g., size, type) and operational state (e.g., orientation, speed). This study seeks to compare these two datasets to relate vessel traffic to the ambient noise a tagged SRKW individual receives.

U.S. guidelines for whale watching have existed in the Salish Sea since 2002, and changes have been made since then to reflect research updates on the effects of whale watching on SRKW [20]. Initially, voluntary guidelines restricted vessels from approaching whales within 100 yards (91 m). In May 2011, federal regulations prohibited vessels from approaching whales within 200 yards (183 m) of whales, or positioning themselves within 400 yards (366 m) of the path of a whale [34]. Research vessels operating under permit are exempt from federal regulations. An additional guideline recommends that vessels do not travel at speeds faster than 7 knots (13 kph) within 400 yd (366 m) of a whale (http://www.bewhalewise.org/). These regulations apply only in U.S. waters. In Canada, whale watching is only subjected to the less stringent voluntary guideline of a 100 m minimum approach distance.

The objectives of this study were to 1) quantify vessel traffic characteristics and activities, 2) estimate the relationship between the quantified vessel characteristics and noise levels received by tagged whales, and 3) assess the relationship between the number of vessels within specific radii of tagged whales and received noise levels. We expected that noise levels would be correlated with vessel characteristics as follows: more noise will be produced by larger vessels with more propellers, traveling at faster speeds and at close distances, where the vessels are parallel to or facing away from the whale. We also expected noise levels to increase with the numbers of vessels in close proximity to tagged whales.

## Methods

### Ethics Statement

Data were collected in U.S. waters under Scientific Research Permit No. 781–1824 and 16163 of the U. S. National Marine Fisheries Service Office of Protected Resources. Data were collected in Canadian waters under Species At Risk Act/Marine Mammal License No. MML 2010-01/SARA-106(B) of the Department of Fisheries & Oceans, Canada. All sampling procedures were reviewed and specifically approved as part of obtaining the field permit. Data collection was also approved by the Northwest Fisheries Science Center's Institutional Animal Care and Use Committee (IACUC).

### Data Collection

Data for this study were collected over three field seasons (September 2010, June 2011, and September 2012) in the semi-enclosed marine waters of the San Juan Archipelago in Washington State, U.S.A. and British Columbia, Canada (approximate range: 48–49°N, 122–123°W, Fig 1). The protected inland waters provide valuable opportunities to access SRKW throughout their core summer habitat while they are also being exposed to high levels of vessel traffic. For each deployment, a DTAG [32] was attached via four suction cups to an individual killer whale with a 7 m carbon fiber pole by an experienced operator on a research vessel. The research vessel was a 6.7 m outboard-motored rigid-hull inflatable with two propellers and a bow pulpit for data collection and tagging. The tags remained on subject whales for an average of 3.6 hours (range: 0.75–7.5 hours) depending on placement of the tag, whale behavior, and the user-specified release time. Twenty-three tags were deployed opportunistically on 22 individual killer whales of varying sex, age, and pod classifications for a total of 82 hours of acoustic data.

The DTAG is an archival tag with two hydrophones that record sound including ambient noise [32]. Depth information is obtained from pressure and temperature sensors incorporated into the tag, allowing pressure to be corrected for temperature [32]. In 2010 and 2011, “version 2” DTAGs were used and in 2012, “version 3” DTAGs were used, but their functionality relative to this study remained consistent (Fig 2). The audio channels of the “version 2” DTAGs had a sampling rate of 192 kHz with 16 bit resolution and the pressure and temperature data were sampled at 50 Hz, and later down-sampled to 5 Hz for calibration and analysis. The audio channels of the “version 3” DTAGs had a sampling rate of 240 kHz with 16 bit resolution and the pressure and temperature data were sampled at 200 Hz, but later down-sampled to the standardized 5 Hz for consistency with data collected by “version 2” tags. A final sampling rate of 5 Hz for pressure and temperature sensor data was deemed to be a sufficient amount of resolution on depth data. Tags were retrieved using a VHF radio signal.

After tags were attached, individual tagged whales were followed from the research boat, as weather allowed, to record vessel traffic characteristics in the vicinity of tagged whales. Surface-based data collection was possible for 20 of the tag deployments (Fig 1). Two laser positioning systems combine a global positioning system (GPS) with built-in data collector to record attribute data (e.g., vessel characteristics), a laser range finder to determine distance, and a compass for bearing to generate geo-referenced (latitude/longitude) data for tagged whales and vessels [35,36]. Data were collected for tagged whales at each surfacing. The research vessel commonly travelled parallel to or behind individual tagged whales at close distances (average: 179 m) in order to obtain accurate and frequent GPS data on subject whales, photo-document the tag’s position on each tagged whale for data calibration purposes, and collect samples (i.e. fecal, prey) opportunistically for objectives of the larger study of SRKW behavioral effects of vessels and associated noise. The following vessel data were recorded: geo-referenced latitude/longitude location, vessel class (commercial and private whale watching, monitoring, enforcement, research, shipping, ferry, military), vessel type (inflatable, small, medium or large hard bottom), vessel position relative to whale (parallel, bow-in perpendicular, bow-out perpendicular), location relative to whales (in front, to the side, behind), and vessel speed based on visual estimation (stationary, slow 0–2 knots, medium 3–4 knots, fast 5–6 knots, and very fast ≥7 knots). For commercial whale watching, research, monitoring, and enforcement vessels, the vessel name was recorded, and later used to obtain data for additional characteristics including the number of propellers, propulsion system (inboard, outboard, Arneson surface drive, jet drive, electric hybrid), and length (m) as provided by the vessel owner. Ideally, data for all vessels within at least 1000 m were collected within 5 minutes, however occasionally data were not recorded for all vessels due to weather conditions, high traffic, or time constraints. In post-processing, custom software was used to calculate the distance between each individual vessel and the surfacing location of the tagged whale that was closest in time to the recorded vessel location [36].

### Data Transformation

Data from the DTAGs were offloaded and unpacked using custom software provided by Woods Hole Oceanographic Institution (WHOI). Data were then calibrated and post-processed using the DTAG toolbox (developed by WHOI) and custom-written routines in Matlab (v. 7.10 and higher). Noise levels from the DTAG audio recordings were measured using criteria similar to those previously published [37]. The key criterion invoked here was exclusion of recording segments that contained whale vocalizations or noise from water flow over the DTAG during whale movements. Noise levels were calculated based on the nominal sensitivities plus gain of the acoustic sensors that were checked with a reference hydrophone. Noise levels based on root-mean-square pressure (in dB re 1 μPa) were integrated over a frequency range of 1–40 kHz. This range is the same as in previous studies and is the relevant range for killer whale communicative signals and hearing sensitivity that overlaps with vessel noise [38,29,31]. Noise levels were initially averaged in 1-second segments to capture the expected variability in noise levels emitted by vessels actively watching killer whales. Depth estimates were also averaged in 1-second segments to temporally match the noise data.

The noise level and vessel traffic datasets were collected on varying temporal and spatial scales due to the differing capabilities of the DTAG computer and human observer to record data, and then matched as well as possible. However, not all audio data were suitable for received noise level estimates. When a suitable noise level was available (i.e. absent of whale vocalizations and flow noise), the time of whale surfacing just prior to, but no more than 5 minutes before, was used as the start of a data interval. All vessel data and 1-second noise level segments recorded within 5 minutes after the identified whale surfacing were included in the data interval. If multiple 1-second noise level segments were available, one average noise level (averaged in pressure then converted to dB) was calculated for the 5-minute interval. If multiple location and behavior attributes were recorded for the same vessel, only the one that occurred closest in time to the relevant whale surfacing event was included.

Numerical vessel characteristics included length, number of propellers, and distance of the individual vessels to tagged whales. A modification was made to the distance measure in order to account for the depth of the whale at the time the noise level was recorded. The average depth of the whale was calculated for each interval and used to calculate the distance from the vessel to the whale at depth using trigonometry.

Categorical vessel characteristics were ordered according to best estimates of their relationship to noise levels (Table 1). Based on previous research, it was assumed that vessels of relatively large sizes and those traveling at relatively high speeds would be louder [39–44]. Vessel orientation was quantified based on two categories of vessel position relative to individual tagged whales (Table 1). Properties of sound propagation are such that the highest received noise levels occur when the vessel motor (i.e. sound source) is facing the receiver (i.e. tagged whale) while the lowest noise levels occur when the vessel motor is directed away from the whale. Other studies have shown that vessel noise is louder when the motor faces the receiver than when the motor faces away from the receiver [41,43,45]. Commercial whale watch vessels vary in the noise levels produced based on their propulsion system [46]. Inboard motors were the loudest, followed by outboard motors and then jet drives [46]. Additional information on electric motors and Arneson surface drives from their manufacturers indicated where on this quantification spectrum they likely fall. Electric motors were expected to be comparable to jet drives, yet quieter than outboard motors while Arneson surface drives were expected to be comparable to outboard motors, but quieter than inboard motors and louder than jet drives. Qualitative attributes for each vessel characteristic were transformed to a numerical rank to permit statistical analysis (Table 1).

**Table 1:** Quantification of vessel characteristics.

Vessel Characteristic	Category	Category 2	Relevance	Rank
Type	Inflatable	N/A	N/A	1
Type	Small Hard Bottom	N/A	N/A	2
Type	Small-Medium Hard Bottom	N/A	N/A	3
Type	Medium Hard Bottom	N/A	N/A	4
Type	Large Hard Bottom	N/A	N/A	5
Speed	Stationary	N/A	N/A	1
Speed	Slow 0–2 knots	N/A	N/A	2
Speed	Medium 3–4 knots	N/A	N/A	3
Speed	Fast 5–6 knots	N/A	N/A	4
Speed	Very Fast 7+ knots	N/A	N/A	5
Orientation	Bow-In perpendicular	Behind whales	Motor away from whale	1
Orientation	Bow-In perpendicular	Side of whales	Motor away from whale	1
Orientation	Bow-In perpendicular	Front of whales	Motor away from whale	1
Orientation	Parallel	Behind whales	Motor away from whale	1
Orientation	Parallel	Side of whales	Motor parallel	2
Orientation	Parallel	Front of whales	Motor facing whale	3
Orientation	Bow-Out perpendicular	Behind whales	Motor facing whale	3
Orientation	Bow-Out perpendicular	Side of whales	Motor facing whale	3
Orientation	Bow-Out perpendicular	Front of whales	Motor facing whale	3
Propulsion system	Jet drive	N/A	N/A	1
Propulsion system	Electric	N/A	N/A	1
Propulsion system	Outboard	N/A	N/A	2
Propulsion system	Arneson surface drive	N/A	N/A	2
Propulsion system	Inboard	N/A	N/A	3

Quantification of vessel characteristics based on categorical qualities collected in the field. For vessel orientation, two field-based categorical qualities were used in conjunction to determine the relevant orientation of the vessel motor relative to the whale.

Only intervals that included data for all characteristics of all the vessels within at least 1000 m of individual tagged whales were included in analyses. Intervals with private whale watching vessels were eliminated from analysis since specific information on their length, number of propellers, and propulsion system was not recorded or available. The remaining intervals also included only whale-oriented vessels with complete data (thereby excluding shipping, ferry, and military vessels). There were 57 intervals of vessel and noise level data. Many intervals included more than one vessel such that there was a total of 112 vessel records in the interval dataset, representing 35 unique vessels. Our research vessel was present in every interval, but the specific groupings of vessels present had characteristics (speed, orientation, distance, etc.) that varied from interval to interval.

The research vessel was the only vessel within 1000 m of the tagged whale in 27 out of the 57 total intervals. The research vessel did not vary in its number of propellers, propulsion system, length, or type, and was most frequently the closest vessel to the whale. As a result, it was possible that including intervals that only represented the research vessel in the statistical analysis could skew the results. Therefore, a separate analysis was conducted excluding any intervals that only included the research vessel. This analysis served to relate noise levels to vessel traffic on a broader scale (e.g. the whale watching fleet), instead of relating noise levels to the characteristics and behavior of the research vessel. There were 30 intervals of vessel and noise level data after research vessel-only intervals were excluded (i.e. when there was at least one other vessel present in addition to the research vessel).

### Modeling Approach

A multiple regression model was developed with the assumption that noise levels in dB relate to each vessel characteristic with a linear relationship. One exception to linearity was the measure of distance between relevant vessels and individual tagged whales. It is estimated that sound propagates in the Salish Sea with transmission loss characterized by spherical spreading [47,6,48]. This led to the assumption that received noise levels would be related to 20 log_10_(distance). In theory vessel power should be proportional to the cube of speed [47], but in practice marine vessel source levels are proportional to speed on a linear scale [44].

Using a maximum likelihood approach, we predicted the noise for each vessel separately with each of the characteristics as predictors. We then summed the predicted noise levels for all the vessels in a given interval to compare to the observed noise level. The equation for the noise level (NL) prediction for all vessels (*V*) of a given interval was:

$${\hat{NL}}_{i}\;=\;20\;{\text{log}}_{10}\;\sum_{v=1}^{V}\;10^{\frac{\beta_{1}+\beta_{2}(length)+\beta_{3}(\#\;propellers)+\beta_{4}(speed)+\beta_{5}(orientation)+\beta_{6}(20\;{\text{log}}_{10}(distance))+\beta_{7}(propulsion\;system)+\beta_{8}(type)}{20}}.$$

For all models, the set of parameters that minimized the negative log likelihood (after omitting constant terms) was found by assuming that error and observed noise levels were normally distributed:

$$-\text{ln}L(NL|\hat{\theta })=-\text{ln}L=\text{ln}\hat{\sigma }+\sum_{i=1}^{n}\;\frac{{\left(N\hat{L}-NL\right)}^{2}}{2{\hat{\sigma }}^{2}}.$$

Parameters were estimated using Solver in Microsoft Excel. We began with the full model and followed a backward stepwise approach using Akaike’s Information Criterion with small-sample correction (AICc) [49] to determine which variable removal most affected the model’s likelihood. We ranked the resulting candidate models according to AICc and used Akaike weights (*w*) to determine relative support for each model [49]. The value of *w* for any model *i* is:

$$w_{i}=\frac{\text{exp}(-0.5\Delta_{i})}{\sum_{r=1}^{R}\;\text{exp}(-0.5\Delta_{r})},$$

where Δ_i_ is the difference in AICc between model *i* and the best model (i.e. lowest AICc) among *R* candidates.

Some categorical vessel characteristics were also analyzed as factors in the negative log likelihood model. These characteristics were type, propulsion system, and orientation. The characteristics were added to the model as factors one at a time. Each characteristic was tested with constrained and non-constrained parameters (see S1 Appendix).

We used model averaging to derive the relationship between vessel traffic and noise levels that was not conditional on any particular model [49]. This method also serves to ameliorate potential effects of uninformative parameters [50]. We applied Akaike weights to the parameters from each model to determine model-averaged parameter estimates:

$$\hat{\bar{\beta }}=\sum_{r=1}^{R}\;w_{i}{\hat{\beta }}_{i}.$$

This allowed the development of a predictive model of noise levels given vessel traffic data for use in other studies. We also used the AICc weights to calculate model-averaged noise level predictions. We compared these predicted values to the observed noise levels to assess model fit. This entire process was repeated for the set of data that did not include research vessel-only intervals.

### Individual Characteristic Analysis

We examined the relationship between received noise levels and the number of vessels within specific radii from the tagged whale without regard to variation in other vessel characteristics. Specific radii included 200 m (minimum distance law in the US enacted in 2011) [34], 400 m (minimum distance law for within the path of the whale), and 1000 m. Information for all characteristics of all vessels within a 5-minute interval was not necessary to determine the number of vessels present. Thus, there were 125 intervals available for these analyses. Linear regression was used to compare noise levels to the count of vessels within each radius. Generalized linear models were also used with a Poisson distribution.

We assessed the relationship between received noise levels and each vessel characteristic individually. For this analysis, each vessel characteristic was averaged for all the vessels of a given interval, using only the 57 intervals of complete data for all vessels present. Linear regression was used to compare noise levels to the following variables separately: vessel length, the number of propellers, vessel speed, vessel orientation, distance of the vessel to the whale, vessel type, and propulsion system. Linear regression was also used to examine the correlation between each of the vessel characteristics. Statistical tests were conducted in the R programming environment [51]. Statistical significance was determined using an assigned alpha level of 0.05.

## Results

### Multiple regression including all intervals

Vessel variables incorporated into the negative log likelihood model as factors resulted in higher AIC_c_ values compared to when these variables were assigned a numerical value (S1 Appendix, S1 Table). Therefore, all model results reported hereafter include vessel type, propulsion system and orientation as ranked variables according to Table 1. The model that best predicted noise levels given the observed data (for all complete intervals, including research vessel-only intervals, n = 57) included vessel length (inverse relationship), number of propellers, and vessel speed (Table 2). This model had an AICc weight of 0.38 indicating substantial weight for alternative models. Models with fewer parameters had very little AICc weight. Models with additional parameters that had substantial AICc weight were those that included distance with an inverse relationship and propulsion system with a positive relationship as expected. The parameter estimates for vessel orientation and type were inversely proportional to noise levels, which was not expected based on their classification. Due to the nature of AICc the additional parameters could be classified as uninformative [50], so model averaging was used to ameliorate the effects.

**Table 2:** Negative log likelihood model results, all intervals.

Model	constant, β_1_	length, β_2_	# propellers, β_3_	speed, β_4_	orientation, β_5_	distance, β_6_	prop system, β_7_	type, β_8_	σ^2^	k	ΔAICc	*w*
Null	98.59	-	-	-	-	-	-	-	7.69	2	**24.05**	<0.01
1	118.15	-2.60	-	-	-	-	-	-	7.09	3	**17.08**	<0.01
2	90.44	-6.87	28.94	-	-	-	-	-	6.36	4	**6.99**	0.01
3	79.63	-4.18	20.84	3.14	-	-	-	-	5.86	5	0	**0.38**
4	80.91	-3.28	19.26	3.12	-2.45	-	-	-	5.75	6	0.39	0.31
5	85.35	-2.35	18.60	3.13	-2.95	-0.21	-	-	5.68	7	1.61	0.17
6	60.04	-2.32	19.83	2.99	-3.30	-0.27	13.14	-	5.63	8	3.27	0.07
7	18.70	-1.49	23.34	2.92	-2.99	-0.25	30.13	-6.24	5.53	9	4.08	0.05

Results of the negative log likelihood model (for all complete intervals, including research vessel-only intervals, n = 57). Model parameters are the vessel characteristics that contribute to received noise. The model that best fit the data was model 3. However, models with additional parameters also had an adequate amount of weight but due to the nature of AICc, could represent uninformative parameters.

**Fig 3:**
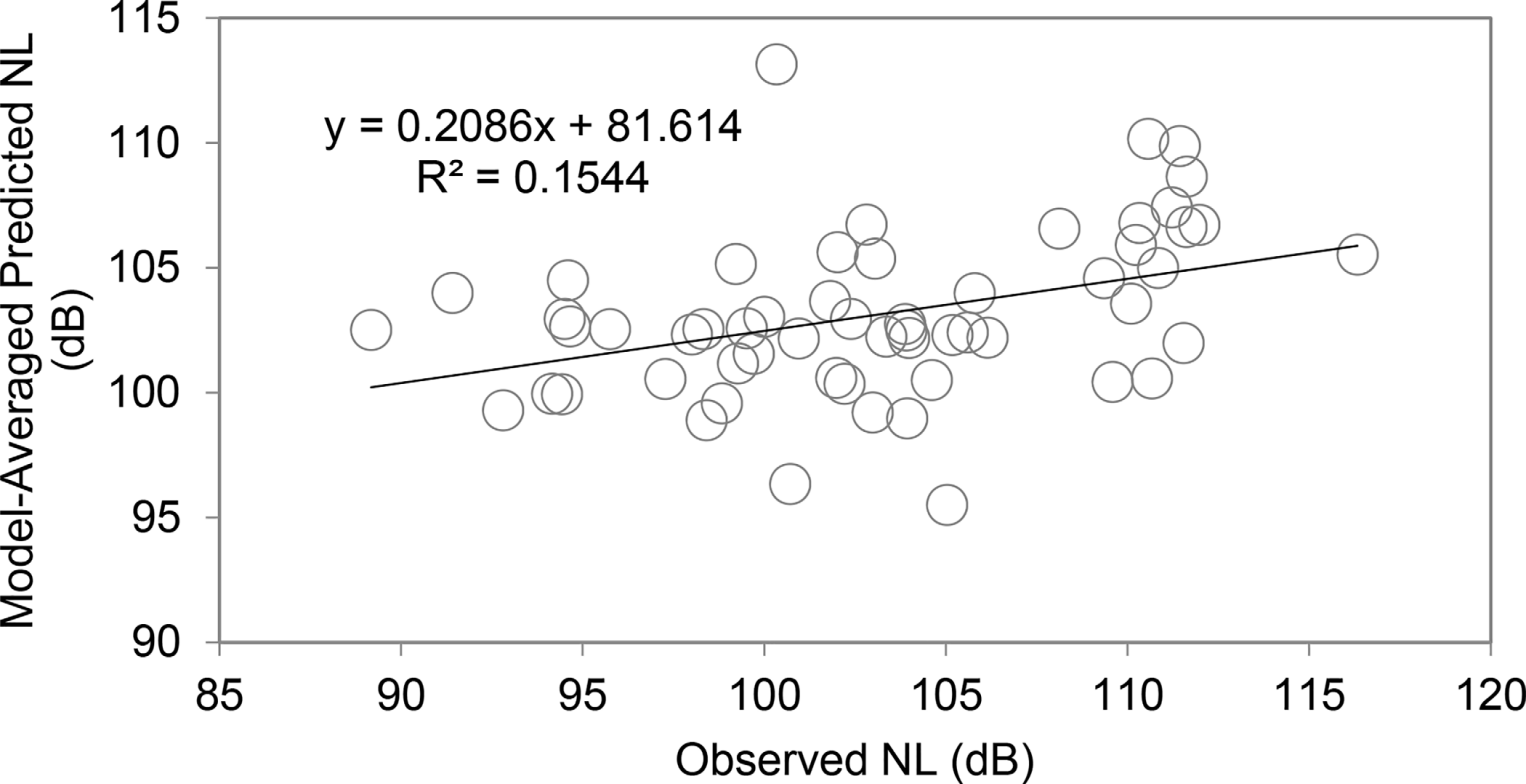
Model predictions vs. observed noise levels, all intervals. Model-averaged predicted noise levels compared to observed noise levels (for all complete intervals, including research vessel-only intervals, n = 57). About 15% of the variation in observed noise levels was explained by the multi-model inference.

Model-averaged predicted noise levels explained approximately 15% of the variation in observed noise levels (Fig 3). Model-averaged parameter estimates indicate that the relationship between noise levels and vessel characteristics can be expressed as:

$$NL={\text{log}}_{10}\sum_{v\;=1}^{V}10^{\frac{76.66-3.34(length)+20.10(\#\;propellers)+3.07(speed)-1.66(orientation)-0.07(20\;{\text{log}}_{10}(distance))+2.47(propulsion\;system)-0.31(type)}{20}}$$

### Multiple regression excluding research vessel-only intervals

The model that best predicted noise levels given the observed data when excluding research vessel-only intervals (n = 30) included only vessel speed as a predictor (Table 3). This model had an AICc weight of 0.45. Models with more parameters than the best model also had an adequate amount of weight. The additional parameters included distance with an inverse relationship and number of propellers, length, and orientation with a positive relationship as expected. Unexpectedly, parameter estimates suggested that vessel type and propulsion system were inversely proportional to noise levels.

**Table 3:** Negative log likelihood model results, excluding research vessel-only intervals.

Model	constant, β_1_	speed, β_4_	# propellers, β_3_	distance, β_6_	type, β_8_	length, β_2_	orientation, β_5_	prop system, β_7_	σ^2^	k	ΔAICc	*w*
Null	93.92	-	-	-	-	-	-	-	6.63	2	**8.61**	<0.01
1	81.38	4.69	-	-	-	-	-	-	5.53	3	0	**0.45**
2	71.62	4.33	5.74	-	-	-	-	-	5.38	4	0.67	0.32
3	79.83	4.14	7.73	-0.24	-	-	-	-	5.32	5	2.38	0.14
4	80.45	4.50	10.52	-0.33	-1.66	-	-	-	5.24	6	3.99	0.06
5	81.52	4.58	8.10	-0.29	-1.98	0.23	-	-	5.23	7	6.50	0.02
6	80.75	4.63	8.01	-0.30	-2.32	0.31	0.70	-	5.23	8	9.16	<0.01
7	85.69	5.05	4.81	-0.21	-2.85	0.60	0.65	-2.74	5.23	9	11.98	<0.01

Results of the negative log likelihood model (excluding research vessel-only intervals, n = 30). Model parameters are the vessel characteristics that contribute to received noise. The model that best fit the data was model 2. However, models with additional parameters also had an adequate amount of weight but due to the nature of AICc, could represent uninformative parameters.

**Fig 4:**
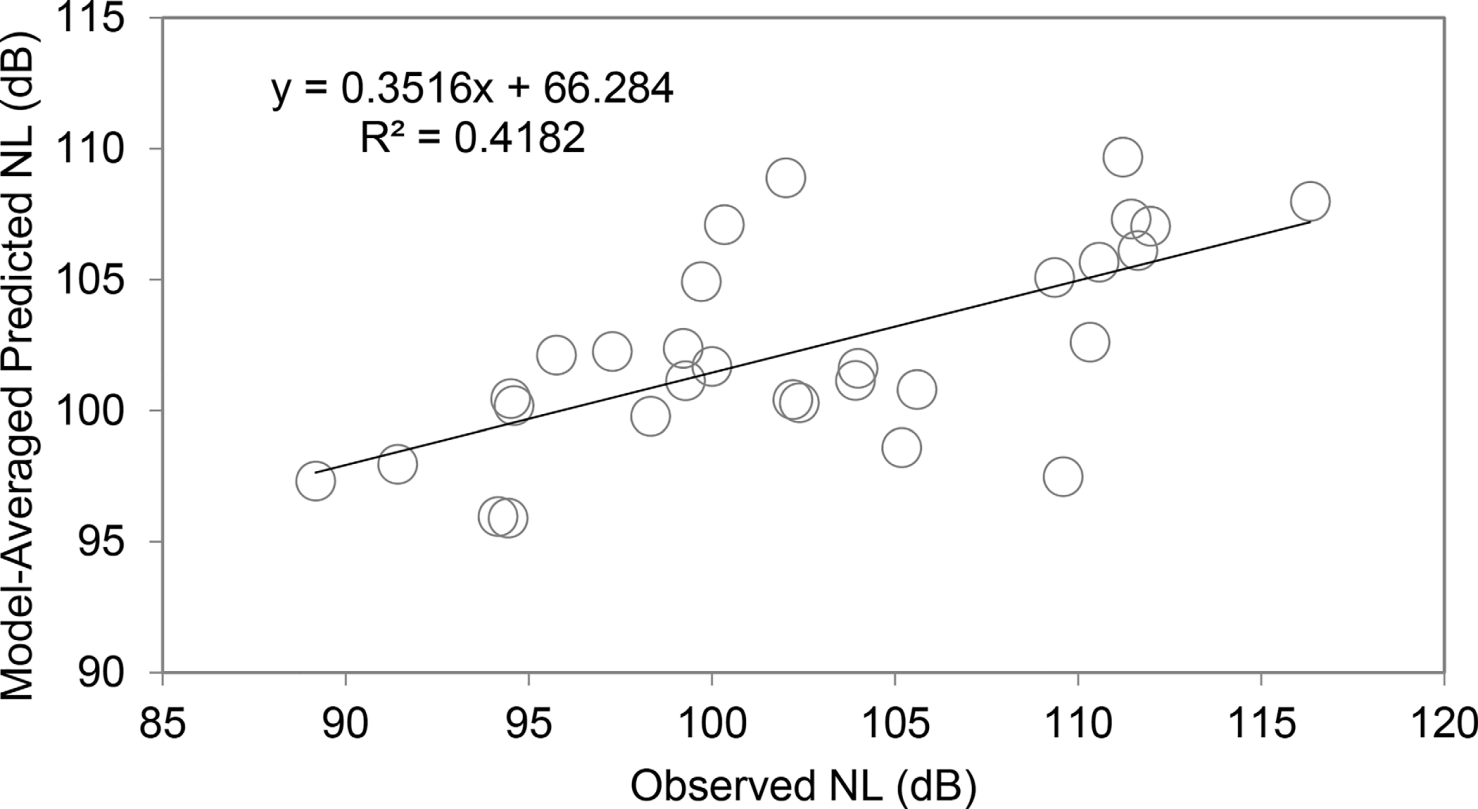
Model predictions vs. observed noise levels, excluding research vessel-only intervals. Model-averaged predicted noise levels compared to observed noise levels (excluding research vessel-only intervals, n = 30). About 42% of the variation in observed noise levels was explained by the multi-model inference.

Model-averaged predicted noise levels when research vessel-only intervals were excluded explained approximately 42% of the variation in observed noise levels (Fig 4). Model-averaged parameter estimates indicate that the relationship between noise levels and vessel characteristics can be expressed as:

$$NL=20{\text{log}}_{10}\sum_{v=1}^{V}\;10^{\frac{78.04+0.006(length)+3.73(\#\;propellers)+4.46(speed)+0.004(orientation)-0.06(20\;{\text{log}}_{10}(distance))-0.003(propulsion\;system)-0.15(type)}{20}}$$

### Individual Characteristic Analysis

Results of the individual linear regression analyses indicated that received noise levels were not correlated with the number of vessels within 200 m (F_1, 123_ = 0.44, p = 0.51), 400 m (F_1, 123_ = 0.28, p = 0.60), or 1000 m (F_1, 123_ < 0.01, p = 0.99). Results of the generalized linear regression with Poisson distribution also indicated that received noise levels were not correlated with the number of vessels within 200 m (Z = 0.40, p = 0.69), 400 m (Z = 0.32, p = 0.75), or 1000 m (Z < 0.01, p = 0.995).

There was no significant relationship between received noise levels and average vessel length (S1 Fig), average distance of vessels to tagged whales (S2 Fig), average vessel orientation (S3 Fig), average vessel type (S4 Fig), or average vessel propulsion system (S5 Fig) per interval. Variation in average vessel length was skewed toward the smaller vessels (S1 Fig). Variation in average vessel distance was slightly skewed toward closer distances (S2 Fig). There was little variation in the average orientation of vessels with most vessels maintaining a parallel orientation while some had motors facing away from individual tagged whales. There were no intervals where on average the vessels had motors facing toward tagged whales (S3 Fig). Variation in average vessel type was heavily skewed toward inflatables and there were no intervals where vessels were on average in the medium or large hard bottom category (S4 Fig). Variation in average vessel propulsion system was quite small with outboard motors present on most vessels per interval (S5 Fig).

**Fig 5:**
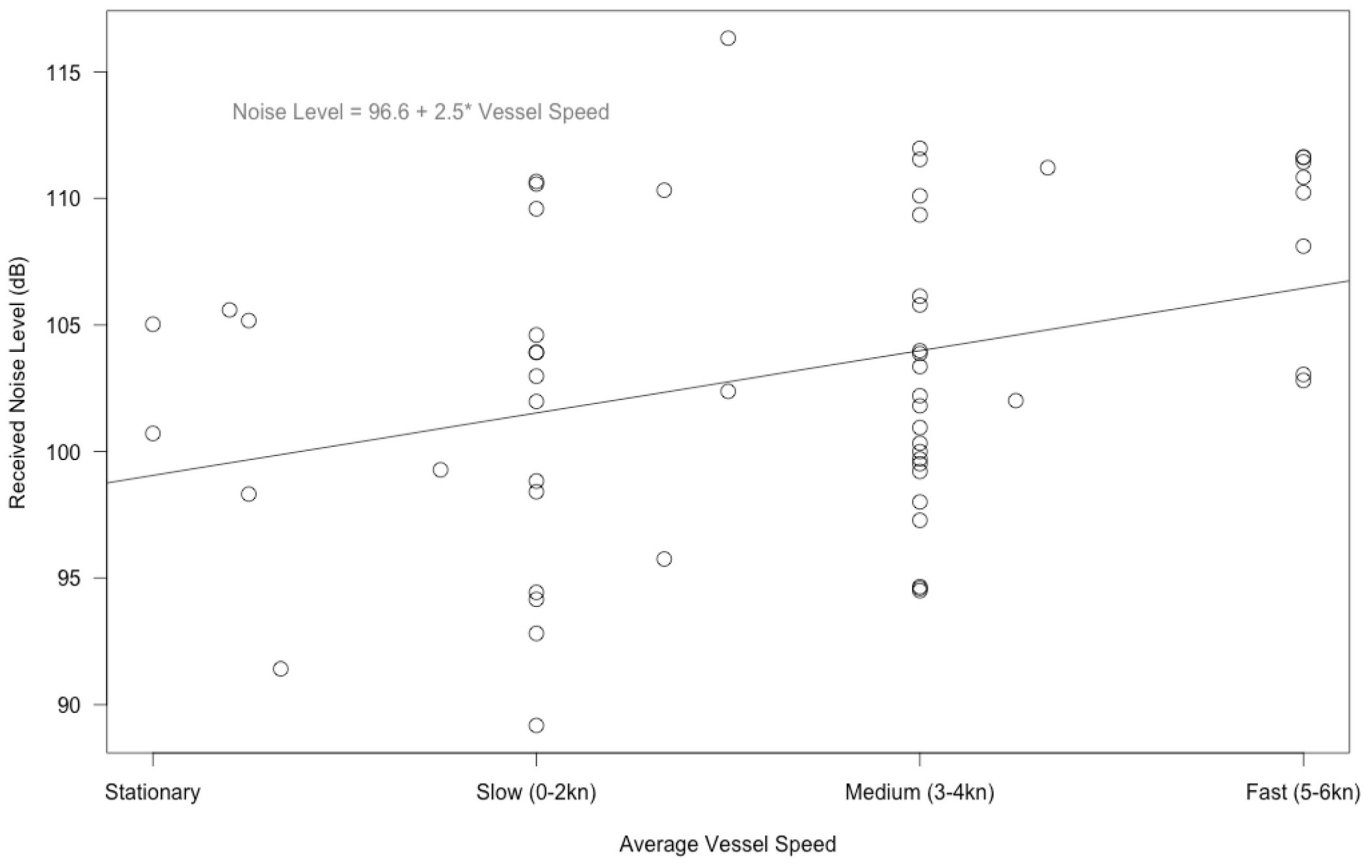
Noise levels and average vessel speed. Received noise levels (dB re 1 μPa) increased significantly with the average vessel speed per interval (F _1, 55_ = 6.704, p = 0.012). There was substantial variation in vessel speed per interval, although no intervals had on average a vessel speed of “Very Fast 7+ knots”.

**Fig 6:**
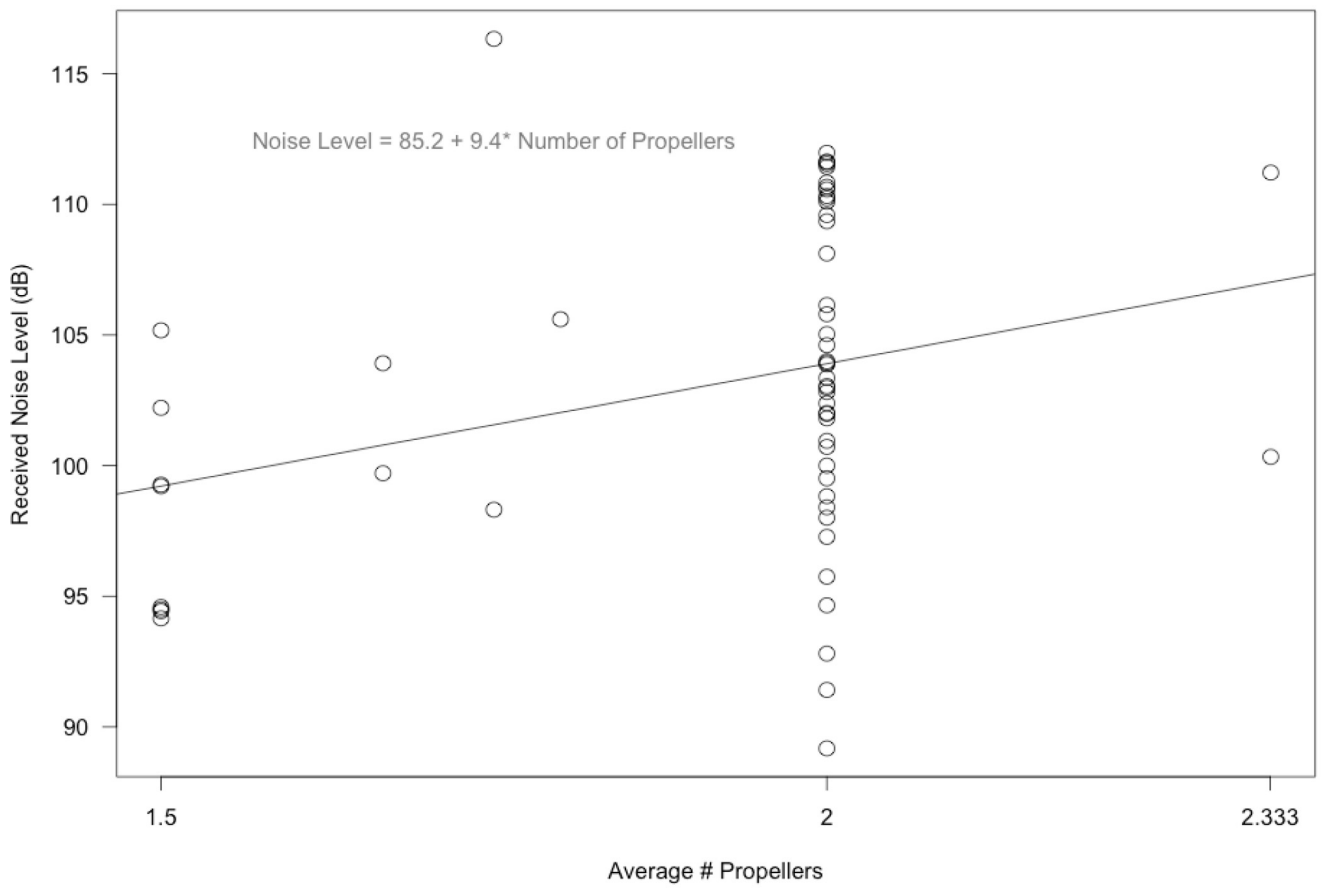
Noise levels and average number of propellers. Received noise levels (dB re 1 μPa) increased significantly with the average number of propellers on the vessels per interval (F1, 55 = 5.476, p = 0.023). There was a lack of variation in the number of propellers among vessels, as most vessels had two propellers. There were occasionally vessels with one or three propellers, but not enough of them to calculate a meaningful average number of vessels of one or three.

Two vessel characteristics, considered separately, were significantly correlated with noise levels even when other variables were not incorporated into the statistical model. Received noise levels increased significantly with the average vessel speed per interval (Fig 5; F_1, 55_ = 6.704, p = 0.012). There was substantial variation in vessel speed per interval, although no intervals had on average a vessel speed of “Very Fast 7+ knots”. Received noise levels also increased significantly with the average number of propellers on the vessels per interval (Fig 6; F_1, 55_ = 5.476, p = 0.023). This was true even though there was a lack of variation in the number of propellers among vessels, as most vessels had two propellers. There were occasionally vessels with one or three propellers, but not enough of them to calculate a meaningful average number of vessels of one or three (Fig 6).

A few vessel characteristics, when averaged within an interval, were correlated with each other. Vessel distance was highly positively correlated with vessel length (F_1, 55_ = 30.62, p<0.001; S6 Fig) and type (F_1, 55_ = 27.77, p<0.001; S7 Fig). The research vessel is clearly visible in the plots as a large number of data points of an inflatable of short length and at close distances to tagged whales. Vessel length was also highly correlated with vessel type (F_1, 55_ = 67.47, p<0.001; S8 Fig). This is an inherent aspect of the characteristics since both are quantifying vessel size in some way. The number of propellers was marginally significantly correlated with vessel speed (F_1, 55_ = 3.385, p = 0.071; S9 Fig).

## Discussion

The significant predictors of noise levels in the likelihood model (including research vessel-only intervals, n = 57) were length (inverse relationship), number of propellers, and vessel speed. While most studies have shown that larger vessels contribute to higher noise levels [39,42], occasionally within a vessel class, length can be inversely proportional to vessel noise for unknown reasons [43]. In this study, it is likely that length was inversely proportional to noise levels because of the highly significant positive correlation between length and distance of relevant vessels to tagged whales (i.e. smaller vessels were more likely to be closer to tagged whales). This might increase the importance of vessel distance as a predictor of noise levels even though it acted as an uninformative parameter in the multi-model inference [49,50]. Model-averaging provided parameter estimates for all vessel characteristics which can be used to predict noise levels in future studies, although it should be noted that relatively little (15%) of the variation in noise levels was explained by the multi-model inference.

The only significant predictor of noise levels in the likelihood model when research vessel-only intervals were excluded (n = 30) was vessel speed. This corroborates the importance of vessel speed as a predictor of noise levels since both models indicated that it was a significant predictor. Model-averaging provided parameter estimates for all vessel characteristics which can also be used to predict noise levels in future studies, and in this case 45% of the variation in noise levels was explained by the multi-model inference. However, observed noise levels had larger variation than predicted noise levels (observed range: 89.2–116.3 dB; predicted range: 95.9–109.7 dB), so interpretation is limited in accurately predicting the lowest and highest noise levels received by individual tagged whales. We suggest that future studies utilize the model-averaged parameters of this model (n = 30) as it removes the potential bias of repeatedly sampling the research vessel, which would have led to a lack of substantial variation in each of the characteristics and inflated the importance of some variables due to the research vessel presence alone. The multi-model inference from this analysis also explains considerably more of the variation in observed noise levels.

Received noise levels were not correlated with the number of vessels within 200 m, 400 m, or 1000 m when other vessel characteristics were disregarded. This is inconsistent with previous research that illustrates that ambient/environmental noise levels (i.e. not those received by tagged whales but measured when the whales were within 400 m) significantly increase with the number of vessels within 1000 m [29]. In the current study, data were collected during periods when vessel traffic was relatively low (the maximum number of vessels was 11), unlike in previous studies [29] where high volumes of commercial whale watching traffic allowed for greater inference from analyses. Analysis of individual characteristics without concurrent regard to other characteristics revealed that received noise levels significantly increased with only two characteristics, the average number of propellers and vessel speed per interval. This further illustrates the importance of the number of propellers as a predictor of noise levels since this characteristic was also a significant predictor of noise levels in the multi-model inference including research vessel-only intervals. Vessel speed is identified as the most important predictor of noise levels as it was a significant predictor in linear regression, in the multi-model inference including research vessel-only intervals, and in the multi-model inference excluding research vessel-only intervals.

The statistical models used in this study were limited in their predictive power due to small sample size. There was a lack of data collected on attributes of private whale watching vessels (i.e. vessel length, number of propellers, and propulsion system) which, based on model results indicating the importance of some of these variables, made it inappropriate to include any intervals where private whale watchers were present in the analysis. As a result, the dataset was limited to a small number of intervals, although other factors also contributed to limitation of sample size. The exclusion of ambient noise levels that included whale vocalizations or flow noise also limited the number of suitable received noise levels used for analysis. Occasional discrepancies in methods of vessel data collection made it difficult to spatiotemporally match vessel and noise level data and also reduced the number of intervals in which all vessels within 1000 m were recorded within 5 min. The 5 min time window was the shortest we could collect all vessel data from surface observations while the received noise levels were averaged in 1 sec segments. Therefore, while the received noise levels can represent a highly dynamic acoustic scene, the data collected from surface observations were limited in temporal resolution.

Predictive power in the statistical methods was also limited by the presence of the research vessel. Repeated measures of the research vessel’s characteristics, which did not vary (i.e. length, number of propellers, propulsion system, and type) or varied infrequently (e.g. orientation and distance), heavily skewed the variation in the characteristics. The research vessel was a small, outboard-motored inflatable with two propellers that frequently travelled parallel to or behind tagged whales at close distances. The lack of variation in these characteristics may explain the inability of the statistical methods to identify significant correlations with received noise levels. Certain vessel characteristics were also highly correlated with each other (i.e. vessel type and length, vessel type and distance, vessel length and distance), decreasing the ability of the statistical methods to separate the effects of different characteristics. Some characteristics could also combine in a way that their effects become non-significant (e.g. large vessels are predicted to produce higher noise levels, but because they occurred farther away from tagged whales, the received noise levels were lower).

There are likely additional factors of vessel traffic that contribute to noise levels received by killer whales that were not included in the dataset. These factors include propeller type, engine horsepower and age, machinery noise, and hull characteristics [52,47,40]. Small-scale changes in vessel behavior, such as turning maneuvers, increase noise levels even after there is a correction for source directionality and speed [41]. Vessels in the dataset, particularly those that were smaller and closer to tagged whales, frequently turned and maneuvered in the highly dynamic whale watch setting, which may contribute to noise level variation not explained by the data collected. There are also abiotic factors that contribute to noise levels received by killer whales that were not included in this dataset including factors affecting sea state [53]. However, data collection could be done only when conditions of weather and sea were relatively mild (i.e. no rain and low wind: Beaufort scale ranged from 0–3 on most days; no vessel data were collected when “white cap” waves were present). Different areas in which data were collected differed in bathymetric characteristics, which can influence sound reflection and absorption. For simplicity such variables were not included in the analysis.

Including additional vessel characteristics and abiotic factors may have improved the predictive power of the statistical methods and models, but the model was still a purposeful representation of the observed data [54]. Noise predictions are complicated and often have substantial shortcomings [55]. However, from this study, it is apparent that vessel speed is one of the most important contributors of noise levels received by killer whales. Other studies have also determined that speed is correlated with vessel noise levels [39–44]. The current management regulations only limit the distance of approach of vessels to endangered SRKW [34], although there is a voluntary guideline to limit vessel speed to less than 7 knots (http://www.bewhalewise.org/). Results from this study will allow managers to assess the effectiveness of current regulations and determine if additional characteristics (e.g. vessel speed) should be formally restricted.

Future studies could address the limitations of the current methodology and apply results to other datasets. For example, substantial data exist on vessel traffic characteristics without concurrent noise level data. Results from the models developed here can be applied to predict the noise levels that Southern Resident killer whales experienced at other times (e.g. during the period of rapid population declined from 1996–2001). Findings could also be applied to other species and study areas where vessel activity may be recorded but access to received noise levels is not possible.

While many studies have examined the effect of vessel characteristics on noise source levels, this is the first study to examine the relationship between vessel characteristics and noise levels received by an endangered whale species or population. Southern Resident killer whales alter their behavior in the presence of vessels and associated noise [23–31]. In the cited studies, the link between vessel traffic characteristics and noise levels actually received by proximate whales is assumed but not explicit. Findings from our study illuminate this relationship and allow for more direct comparisons between vessels and received noise. Other studies have focused on relating environmental noise to vessel variables, but these studies are limited to large ships as the important variable data (e.g., type, length, speed over ground) are obtained from AIS information [44,45]. Without the present study, there is limited empirical evidence of the contributions that smaller vessels and their variables have on noise. However, smaller vessels occur regularly in coastal habitats particularly during the summer months, which overlap temporally with some marine mammal populations. Finally, acoustic tags have been used extensively to examine vocal and movement behavior of marine mammals [33]. Our study illustrates a new use of the tag technology, applicable to other studies where human use of the environment is measured concurrently with animal behavior.

## Supporting Information

S1 Appendix Analysis of qualitative characteristics as factors. The qualitative vessel characteristics: type, orientation and propulsion system, were also analyzed as factors in the negative log likelihood model.
(PDF)

S2 Appendix Underlying data for analyses. Spreadsheet includes: whale and vessel locations, vessel characteristics, and received noise levels for all intervals in this study.
(XLSX)

S1 Fig Noise levels and average vessel length. There was no significant relationship between received noise levels (dB re 1 μPa) and average vessel length (m) per interval. Variation in average vessel length was skewed toward the smaller vessels.
(TIF)

S2 Fig Noise levels and average distance of vessels to tagged whales. There was no significant relationship between received noise levels (dB re 1 μPa) and the average distance of vessels to tagged whales (m) per interval. Variation in average vessel distance was slightly skewed toward closer distances.
(TIF)

S3 Fig Noise levels and average vessel orientation. There was no significant relationship between received noise levels (dB re 1 μPa) and the average vessel orientation per interval. Orientation descriptions are relating the motor’s relationship to the whale (i.e. motor away indicates the motor is facing away from the whale, see Table 1). There was little variation in the average orientation of vessels with most vessels maintaining a parallel orientation while some had motors facing away from the whale. There were no intervals where on average the vessels had motors facing toward the whale.
(TIF)

S4 Fig Noise levels and average vessel type. There was no significant relationship between received noise levels (dB re 1 μPa) and the average vessel type per interval. Variation in average vessel type was heavily skewed toward inflatables and no intervals where vessels were on average of the medium or large hard bottom distinction.
(TIF)

S5 Fig Noise levels and average propulsion system. There was no significant relationship between received noise levels (dB re 1 μPa) and the average vessel propulsion system per interval. Variation in average vessel propulsion system was very poor with outboard motors present on most vessels per interval.
(TIF)

S6 Fig Average distance of vessels to tagged whales and average vessel length. The average distance (m) of vessels to tagged whales had a highly significant correlation with average vessel length (m) per interval (F_1, 55_ = 30.62, p<0.001).
(TIF)

S7 Fig Average distance of vessels to tagged whales and average vessel type. The average distance (m) of vessels to tagged whales had a highly significant correlation with average vessel type per interval (F_1, 55_ = 27.77, p<0.001).
(TIF)

S8 Fig Average vessel length and average vessel type. The average vessel length (m) had a highly significant correlation with average vessel type per interval (F_1, 55_ = 67.47, p<0.001).
(TIF)

S9 Fig Average number of propellers and average vessel speed. The average number of propellers had a marginally significant correlation with average vessel speed per interval (F_1, 55_ = 3.385, p = 0.071).
(TIF)

S1 Table AICc results of models with qualitative characteristics as factors. Negative log likelihood model results when vessel type, propulsion system and orientation were included as factors. The AICc value for the full model excluding research vessel-only intervals where each qualitative characteristic was assigned a numerical value (according to Table 1) was 151.05.
(DOCX)
