# Supplementary material for: The Relationship between Vessel Traffic and Noise Levels Received by Killer Whales (Orcinus orca)
Source: PLoS One. 2015 Dec 2;10(12):e0140119. doi: 10.1371/journal.pone.0140119 (PMC4667929; doi:10.1371/journal.pone.0140119)
Supplement: S1 Appendix — The qualitative vessel characteristics: type, orientation and propulsion system, were also analyzed as factors in the negative log likelihood model. (PDF) [file pone.0140119.s001.pdf]

S1 Appendix. Some vessel characteristics were also analyzed as factors in the negative log likelihood model. These characteristics were type, propulsion system, and orientation. The characteristics were added to the model as factors one at a time. Each characteristic was tested with constrained and non-constrained parameters. In all cases, the AICc was higher than it was for the original model where each qualitative characteristic was assigned a numerical value. Therefore, using vessel characteristics quantified as numeric values in Table 1 improved the relative quality of the statistical analyses conducted in this study.
