# Supplementary figures and images for: The Relationship between Vessel Traffic and Noise Levels Received by Killer Whales (Orcinus orca)
Source: PLoS One. 2015 Dec 2;10(12):e0140119. doi: 10.1371/journal.pone.0140119 (PMC4667929; doi:10.1371/journal.pone.0140119)

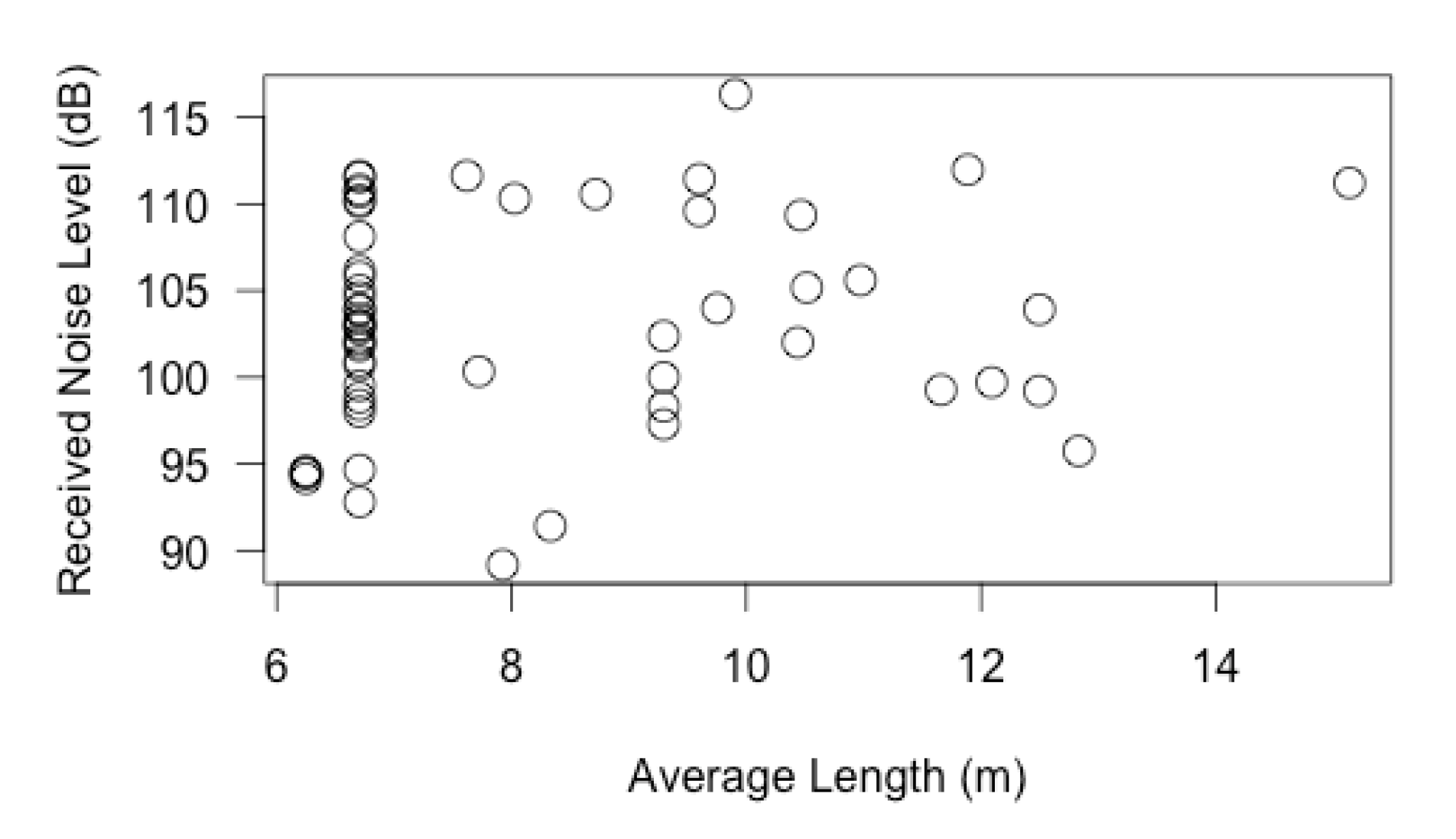

Supplement: S1 Fig — There was no significant relationship between received noise levels (dB re 1 μPa) and average vessel length (m) per interval. Variation in average vessel length was skewed toward the smaller vessels. (TIF) [file pone.0140119.s003.tif]

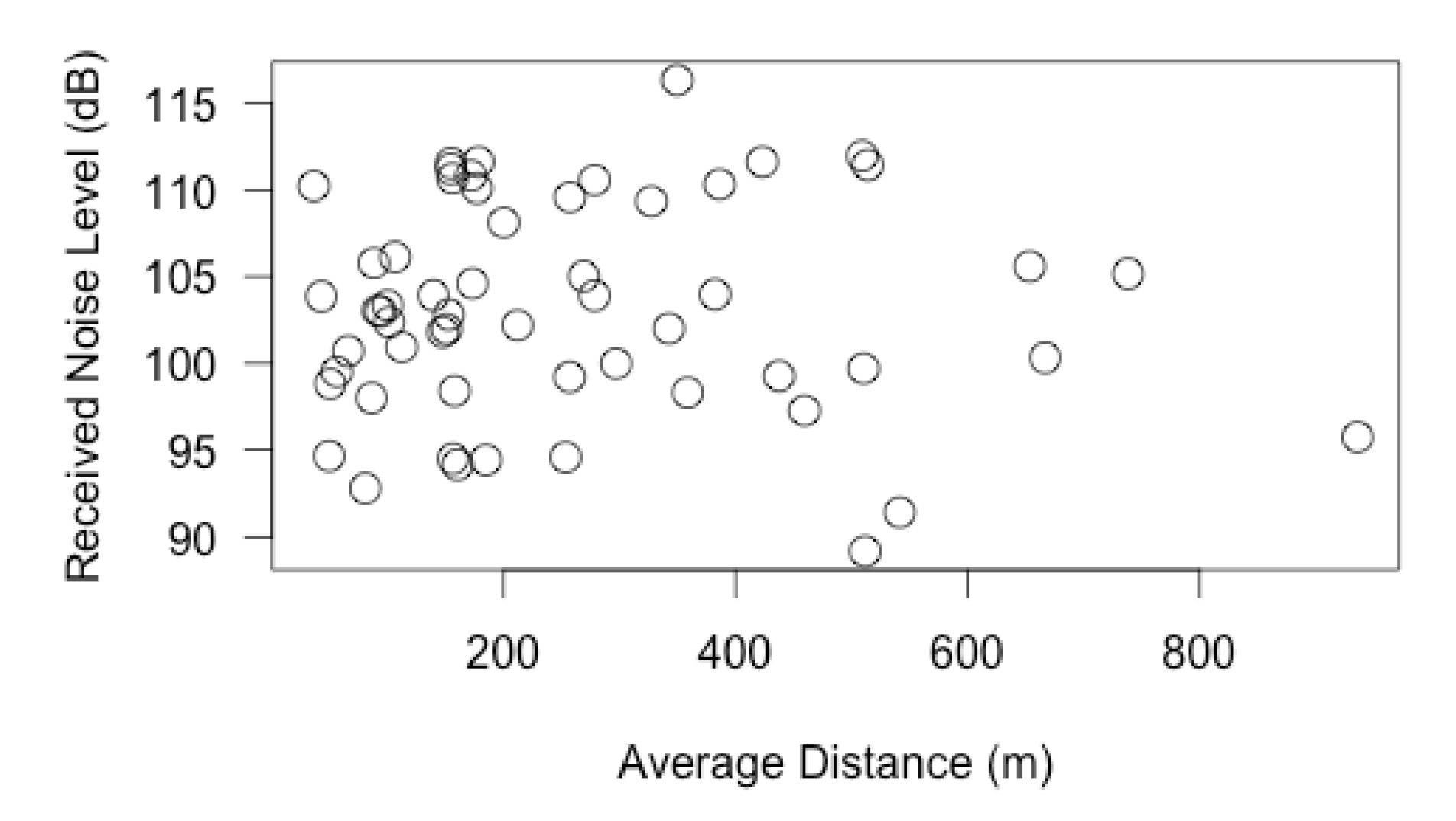

Supplement: S2 Fig — There was no significant relationship between received noise levels (dB re 1 μPa) and the average distance of vessels to tagged whales (m) per interval. Variation in average vessel distance was slightly skewed toward closer distances. (TIF) [file pone.0140119.s004.tif]

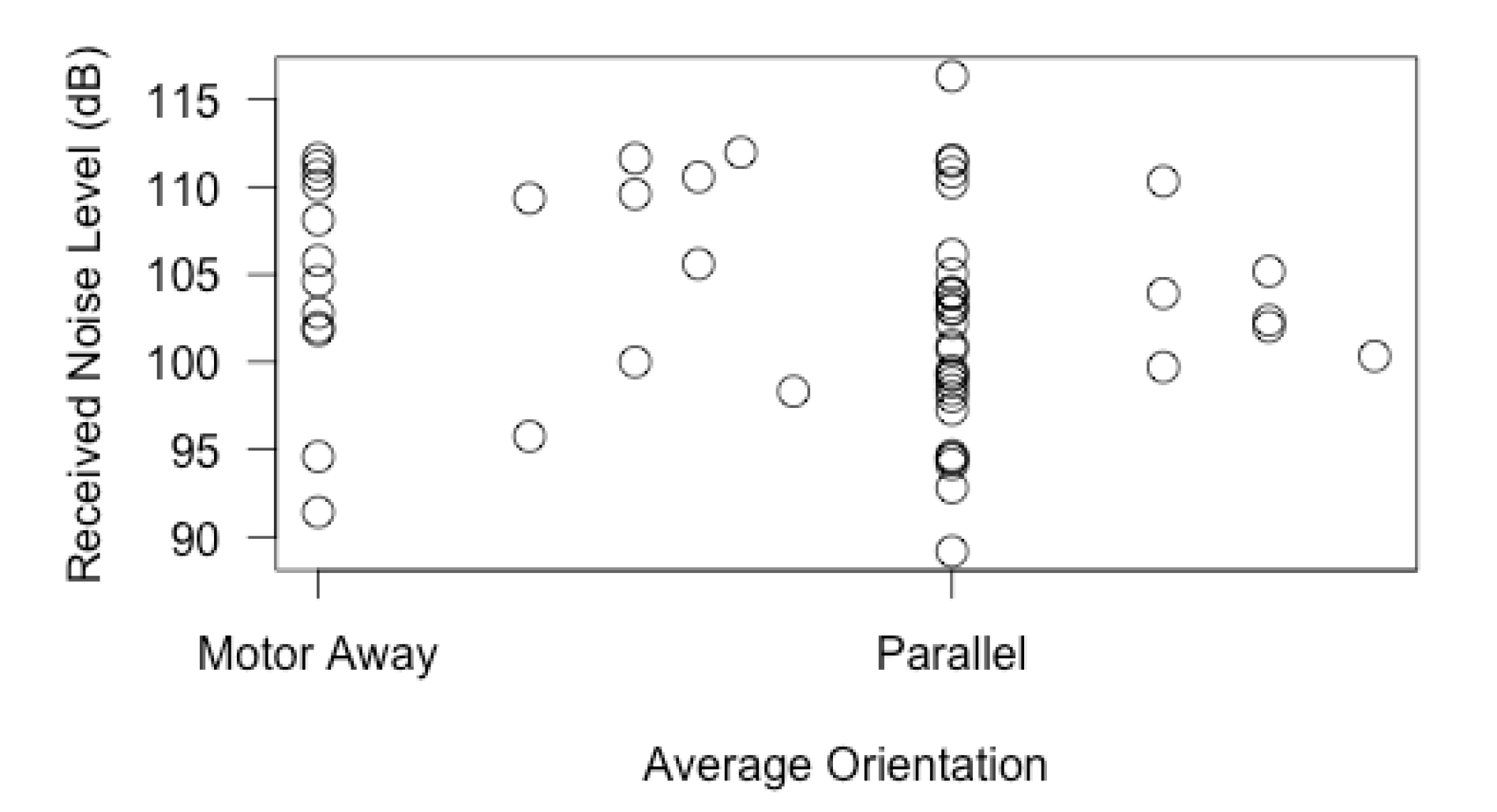

Supplement: S3 Fig — There was no significant relationship between received noise levels (dB re 1 μPa) and the average vessel orientation per interval. Orientation descriptions are relating the motor’s relationship to the whale (i.e. motor away indicates the motor is facing away from the whale, see Table 1). There was little variation in the average orientation of vessels with most vessels maintaining a parallel orientation while some had motors facing away from the whale. There were no intervals where on average the vessels had motors facing toward the whale. (TIF) [file pone.0140119.s005.tif]

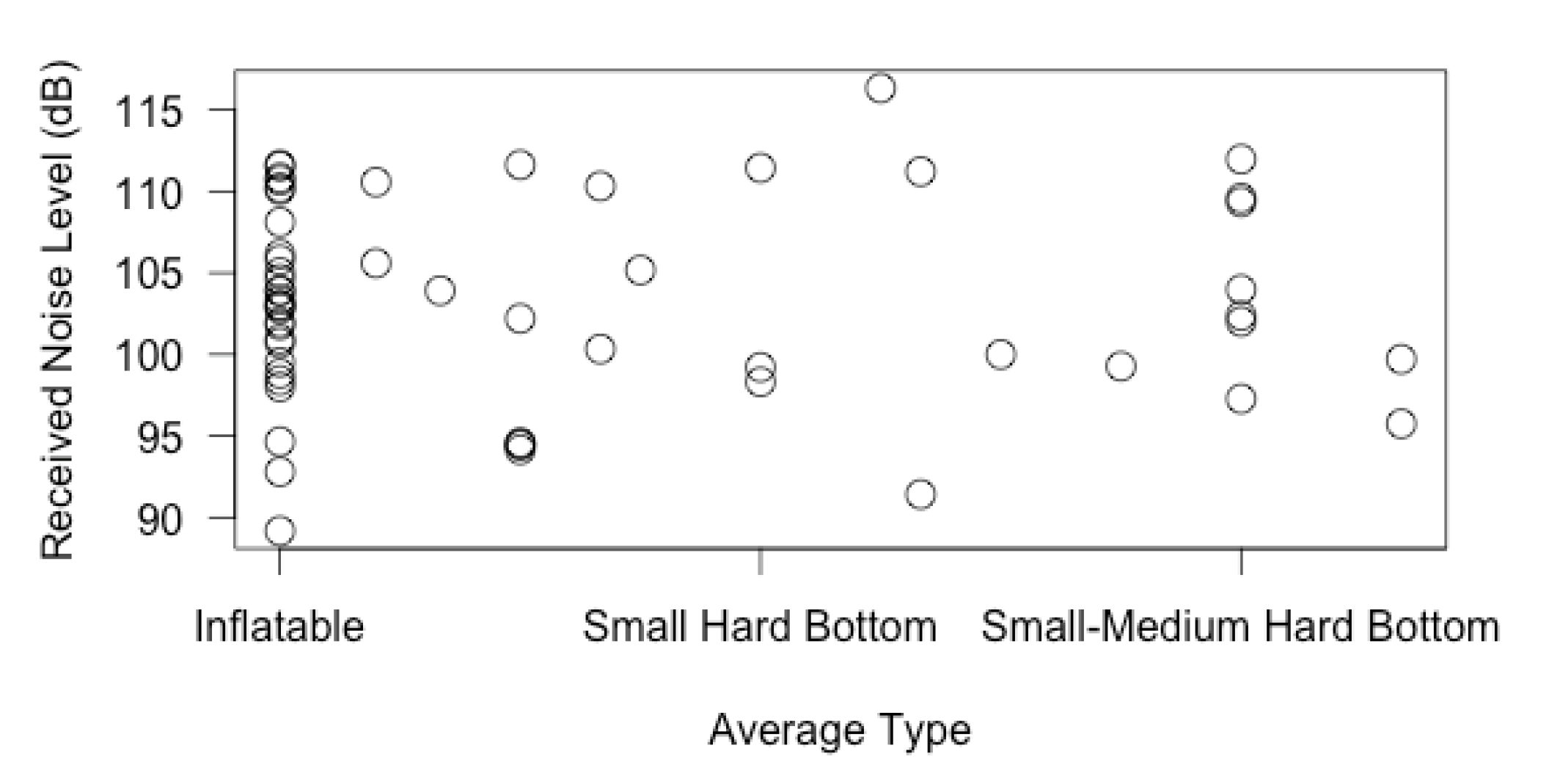

Supplement: S4 Fig — There was no significant relationship between received noise levels (dB re 1 μPa) and the average vessel type per interval. Variation in average vessel type was heavily skewed toward inflatables and no intervals where vessels were on average of the medium or large hard bottom distinction. (TIF) [file pone.0140119.s006.tif]

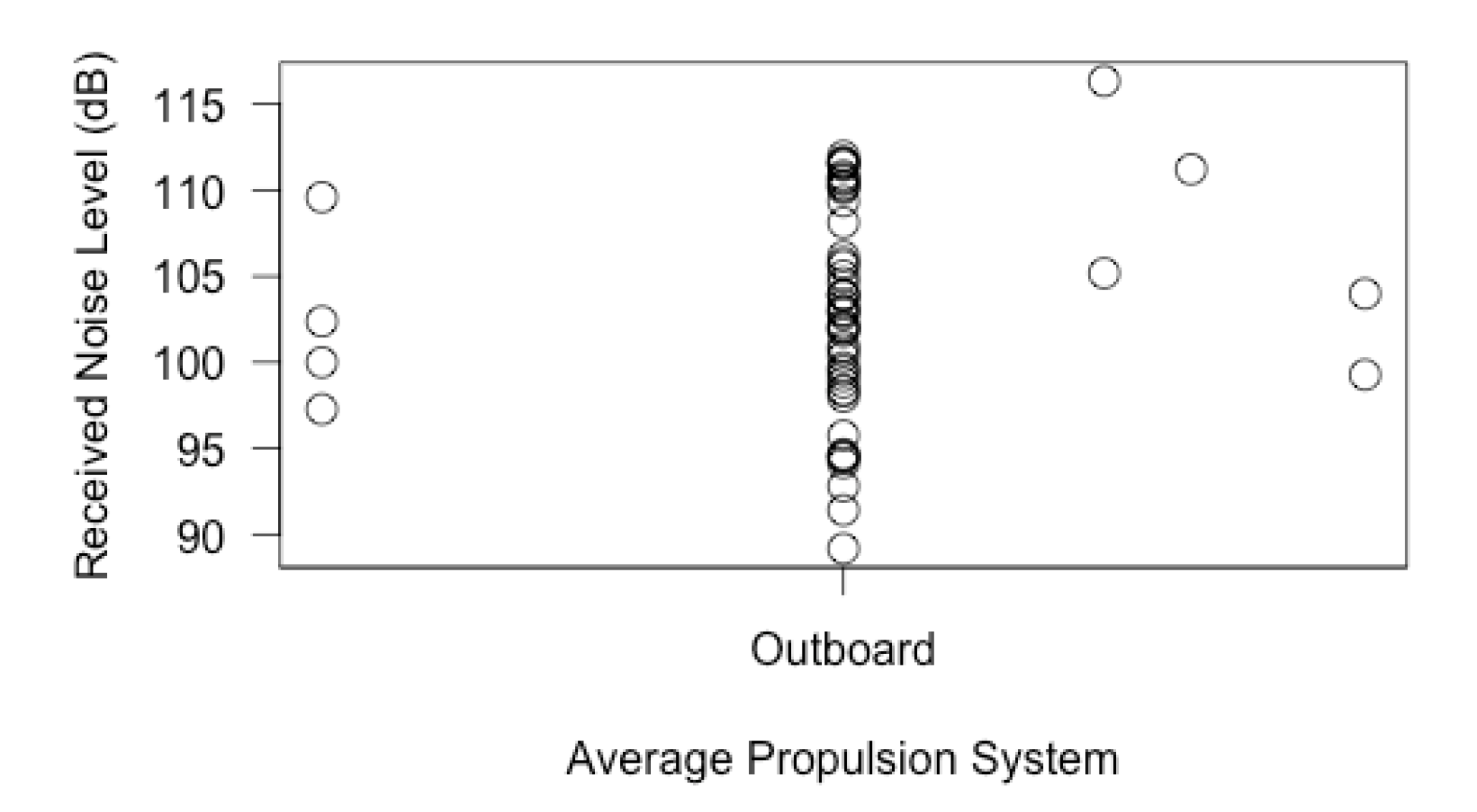

Supplement: S5 Fig — There was no significant relationship between received noise levels (dB re 1 μPa) and the average vessel propulsion system per interval. Variation in average vessel propulsion system was very poor with outboard motors present on most vessels per interval. (TIF) [file pone.0140119.s007.tif]

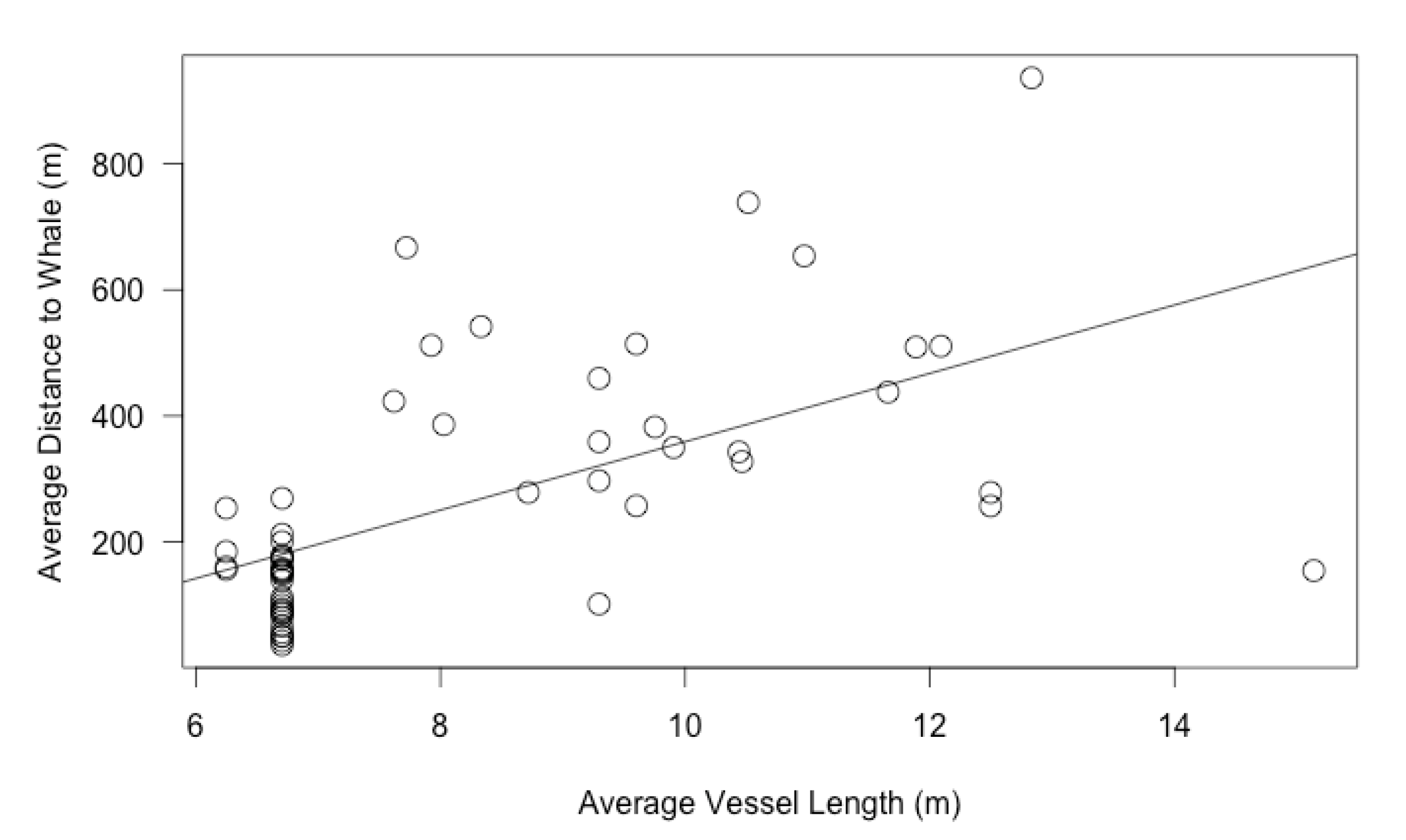

Supplement: S6 Fig — The average distance (m) of vessels to tagged whales had a highly significant correlation with average vessel length (m) per interval (F1, 55 = 30.62, p<0.001). (TIF) [file pone.0140119.s008.tif]

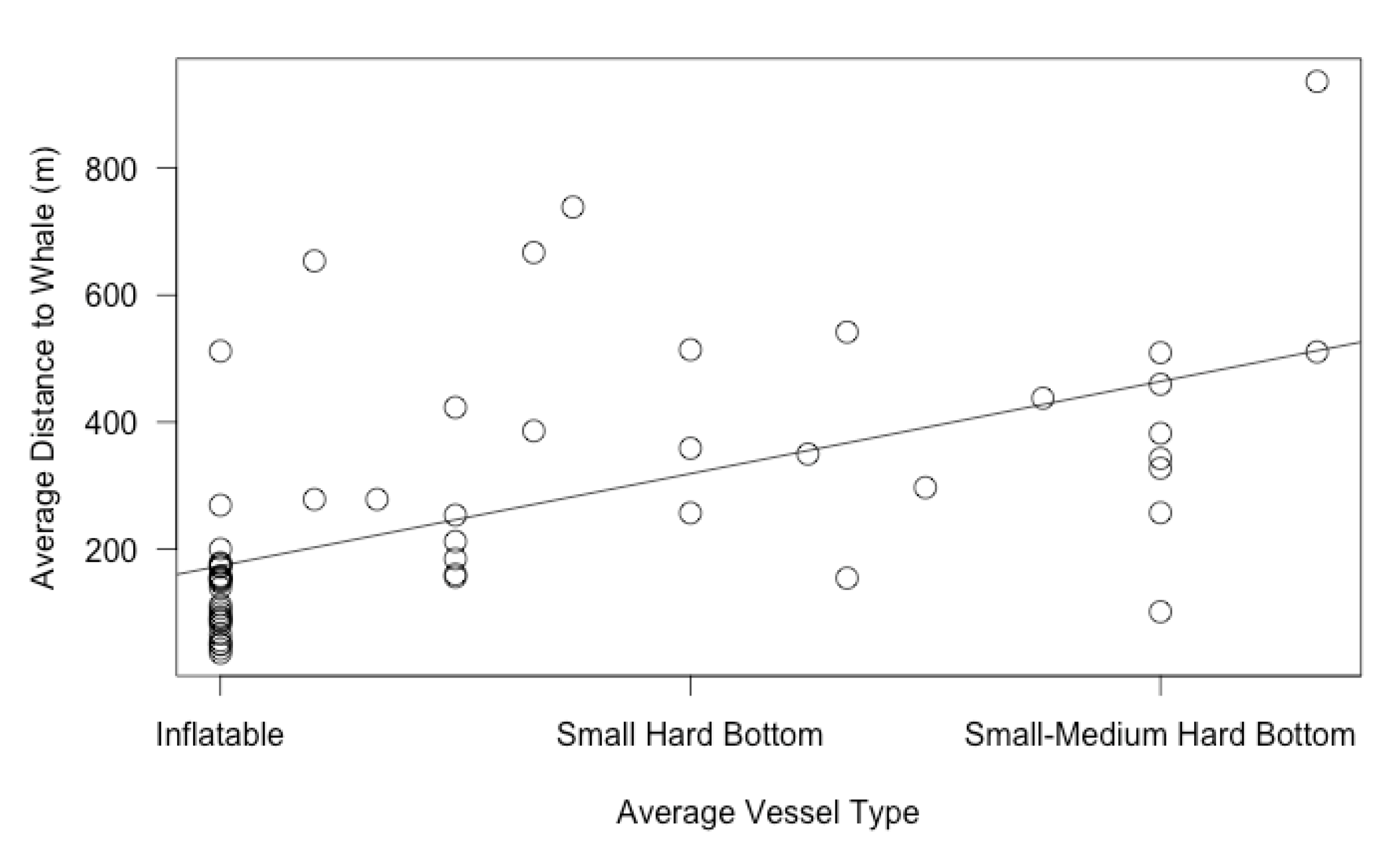

Supplement: S7 Fig — The average distance (m) of vessels to tagged whales had a highly significant correlation with average vessel type per interval (F1, 55 = 27.77, p<0.001). (TIF) [file pone.0140119.s009.tif]

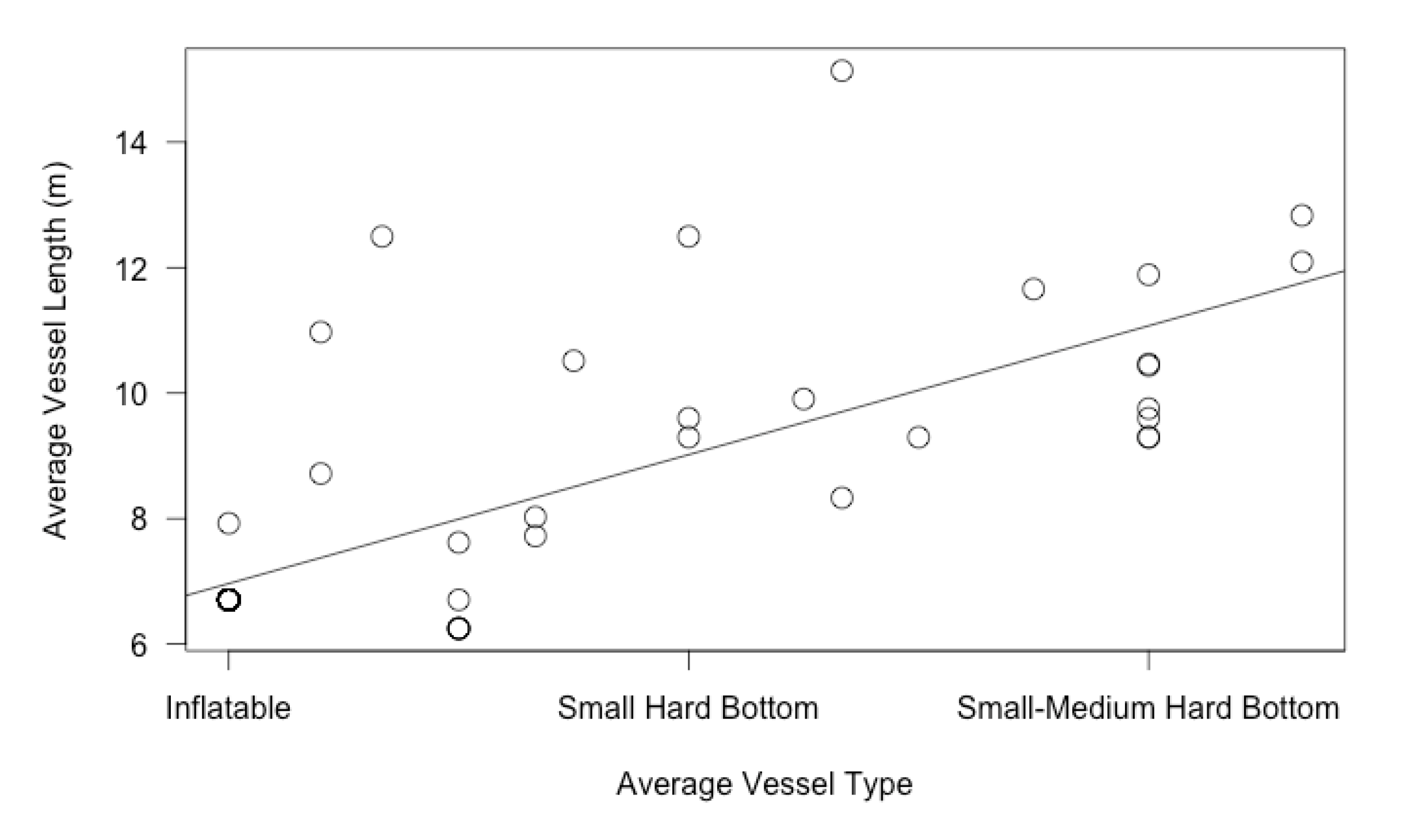

Supplement: S8 Fig — The average vessel length (m) had a highly significant correlation with average vessel type per interval (F1, 55 = 67.47, p<0.001). (TIF) [file pone.0140119.s010.tif]

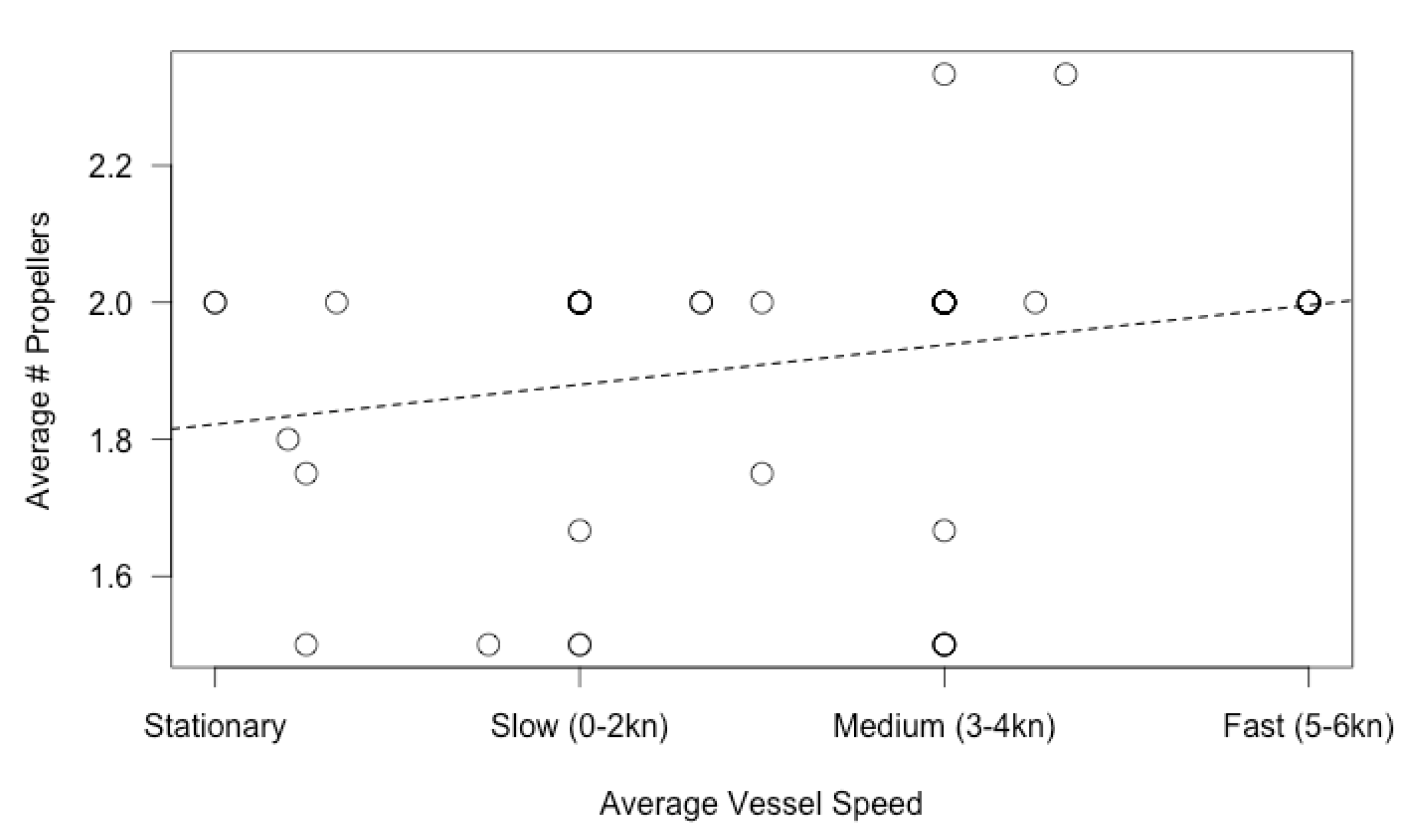

Supplement: S9 Fig — The average number of propellers had a marginally significant correlation with average vessel speed per interval (F1, 55 = 3.385, p = 0.071). (TIF) [file pone.0140119.s011.tif]
