# Supplementary material for: The Relationship between Vessel Traffic and Noise Levels Received by Killer Whales (Orcinus orca)
Source: PLoS One. 2015 Dec 2;10(12):e0140119. doi: 10.1371/journal.pone.0140119 (PMC4667929; doi:10.1371/journal.pone.0140119)
Supplement: S1 Table — Negative log likelihood model results when vessel type, propulsion system and orientation were included as factors. The AICc value for the full model excluding research vessel-only intervals where each qualitative characteristic was assigned a numerical value (according to Table 1) was 151.05. (DOCX) [file pone.0140119.s012.docx]

S1 Table.

| Vessel Characteristic as factor | AICc value |
| --- | --- |

|  | Constrained | Not-constrained |
| --- | --- | --- |
| Type | 164.30 | 159.52 |
| Propulsion System | 157.08 | 156.68 |
| Orientation | 156.77 | 152.46 |

Negative log likelihood model results when vessel type, propulsion system and orientation were included as factors. The AICc value for the full model excluding research vessel-only intervals where each qualitative characteristic was assigned a numerical value (according to Table 1) was 151.05.
